# Surgical Options for Resectable Lung Adenosquamous Carcinoma: A Propensity Score-Matched Analysis

**DOI:** 10.3389/fonc.2022.878419

**Published:** 2022-07-01

**Authors:** Shuncang Zhu, Tao Ge, Yicheng Xiong, Jing Zhang, Di Zhu, Liangdong Sun, Nan Song, Peng Zhang

**Affiliations:** Department of Thoracic Surgery, Shanghai Pulmonary Hospital, Tongji University School of Medicine, Shanghai, China

**Keywords:** lobectomy, lung adenosquamous carcinoma, propensity score-matched, sublobar resection, survival analysis

## Abstract

**Background:**

Surgery is the primary treatment option for Lung adenosquamous carcinoma (ASC) patients. However, no study compares the benefits of lobectomy and sublobar resection in ASC patients.

**Methods:**

A total of 1379 patients in the Surveillance, epidemiology, and End Results (SEER) database and 466 patients in Shanghai Pulmonary Hospital (SPH) were enrolled. Survival benefits were evaluated after possible confounders were eliminated by propensity score matching (PSM).

**Results:**

After 1:3 PSM, 463 SEER database patients and 244 SPH patients were enrolled. Lobectomy was associated with better overall survival (OS) and disease-free survival (DFS) than sublobar resection for ASC patients (5-year OS of SEER: 46.9% vs. 33.3%, *P* =0.017; 5-year OS of SPH: 35.0% vs. 16.4%, *P* =0.002; 5-year DFS of SPH: 29.5% vs. 14.8%, *P* =0.002). Similar results were observed in stage I patients. Univariate and multivariate Cox regression analyses showed that sublobar resection was an adverse prognostic factor independently (SEER: HR: 1.40, 95%CI: 1.08-1.81, *P* =0.012; SPH: HR: 1.73, 95%CI: 1.11-2.70, *P* =0.015). Subgroup analysis showed that all of the ASC patient subtypes tended to benefit more from lobectomy than sublobar resection.

**Conclusions:**

Lobectomy remains the primary option for ASC patients compared to sublobar resection, including stage I.

## 1 Introduction

Lung adenosquamous carcinoma (ASC) is a relatively rare pathological type of lung cancer, accounting for about 0.4%-4.0% (1). In 1999, the WHO histological classification of lung and pleural neoplasms first defined the diagnostic criteria for ASC as a carcinoma showing components of both adenocarcinoma (AC) and squamous cell carcinoma (SCC), with each comprising at least 10% of the tumor. This definition continues to the 2021 WHO Classification of Lung Tumors (2). However, ASC is not a simple mixture of AC and SCC, with unique biological and clinical characteristics. Compared with pure AC or SCC, ASC has more vital aggressiveness and poor prognosis (3–5). Currently, surgery is the primary treatment option for ASC patients, similar to other non-small cell lung cancer (NSCLC) treatments (6). Combined treatment with chemotherapy can improve the survival rate of patients and reduce the risk of distant metastasis in patients with resectable ASC (5).

For a long time, lobectomy represents a relatively safe surgical option for lung cancer that reduces the risk of local recurrence and improves overall survival (7, 8). But a growing number of studies (9–11) suggested that sublobar resection appears to achieve the same level of efficacy and accuracy, with benefits for preserving lung function and reducing the incidence of perioperative complications. Therefore, the superiority of lobectomy versus sublobar resection is still controversial.

To the best of our knowledge, few studies are concerned with which type of surgery provides more significant survival benefits for ASC patients. To address this unresolved issue, we retrospectively analyzed the survival impact of different surgical procedures (lobectomy and sublobar resection) on ASC patients from the Surveillance, Epidemiology, and End Results (SEER) database and Shanghai Pulmonary Hospital (SPH) in China.

## 2 Methods

### 2.1 Patients and Data Collection

#### 2.1.1 SEER Database Cohort

**Figure 1:**
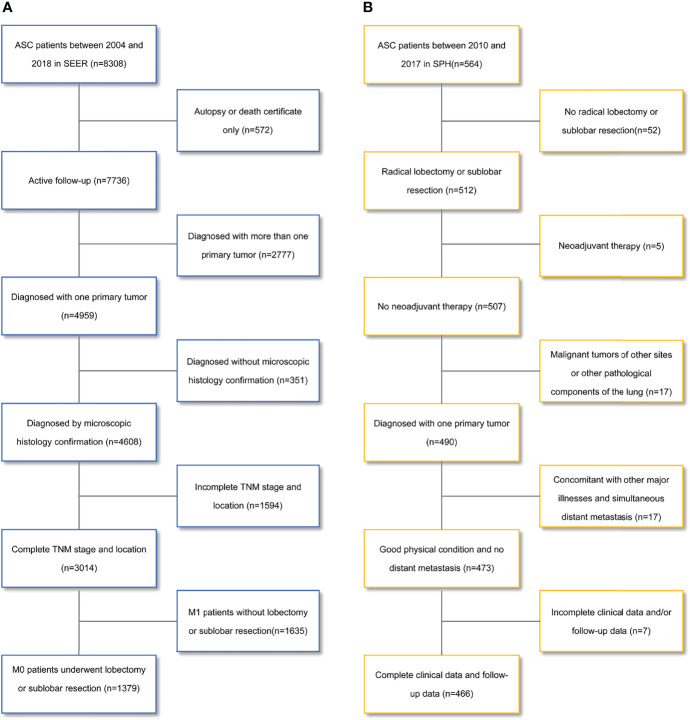
Flowchart for patient selection. **(A)** the Surveillance, Epidemiology, and End Results database; **(B)** the Shanghai Pulmonary Hospital database. ASC, lung adenosquamous carcinoma.

One cohort in this retrospective study was enrolled from the SEER database. 8308 patients diagnosed with ASC between 2004 and 2018 were extracted from SEER*Stat software (version 8.3.9). ASC was defined based on the third edition of the International Classification of Diseases for Oncology (ICD-O-3) with site code C34.0-C34.9 and histological type code 8560/3. We reclassified the TNM stage according to the eighth edition of the American Joint Committee on Cancer (AJCC) TMN stage. Exclusion criteria included: unknown TNM stage, no diagnosis by histology, M1 stage, and other surgery except lobotomy or sublobar resection, diagnosed with more than one primary tumor. **Figure 1A** shows the screening process. Ultimately, 1379 cases were included in the cohort.

#### 2.1.2 SPH Cohort

We also selected 564 patients with ASC confirmed by postoperative pathology from the patient database of SPH between 2010 and 2017. The exclusion criteria were: no radical lobectomy or sublobar resection, neoadjuvant therapy, malignant tumors of other sites or other pathological components of the lung, concomitant with other major illnesses, simultaneous distant metastasis, and incomplete clinical data and/or follow-up data. **Figure 1B** shows the screening process. Finally, 466 patients were enrolled in the cohort.

This study was conducted following the Declaration of Helsinki (revised in 2013) and obtained the consent of the Institutional Review Board of Shanghai Pulmonary Hospital.

### 2.2 Operation and Follow-Up

Surgical methods were classified into lobectomy and sublobar resection (i.e., wedge and segmental resection). In SPH, Lobectomy or sublobar resection was determined by each surgical team in the thoracic surgery department and was reported and discussed at a daily morning meeting. Sublobar resection was generally recommended as a priority for patients with old age, impaired lung function, a higher risk of lobectomy, or early-stage NSCLC with a tumor diameter ≤2 cm. In the first two years after surgery, patients were required to receive a routine physical examination every 3 to 6 months, including physical examination, serum tumor markers, chest CT scans, abdominal ultrasound. And then once a year from the third to the fifth year. Brain magnetic resonance imaging, bone scans, and PET-CT should be performed only when recurrence or metastasis is suspected. We followed up with patients’ medical records or phone calls to record their survival or disease progression. The follow-up deadline for the SEER database cohort was December 31, 2020, and for the SPH cohort was July 1, 2021.

### 2.3 Statistical Analysis

Based on different surgical methods, the eligible population was divided into lobectomy and sublobar resection groups. The chi-square test or Fisher’s exact test was used to analyze categorical variables. Continuous variables were compared using Student’s T-test or Mann-Whitney U-test. A 1:3 propensity score matching (PSM) was performed to reduce confounding factors in the baseline information. The match was conducted using the nearest-neighbor algorithm. The caliper width was 0.005 in the SEER database. Propensity scores were calculated using logistic regression with the following covariates: age, sex, location, Pathologic T descriptor, Pathologic N descriptor, lymph node dissection (LND), radiation, and chemotherapy. And the race was also calculated in the SEER database cohort. *P >*0.05 was regarded as an acceptable balance. All subsequent statistical analyses were based on PSM results.

Overall survival (OS) was defined as the time from diagnosis to death and Disease-free survival (DFS) was defined from diagnosis to the first event (recurrence, metastasis, or death). Follow-up time was calculated from diagnosis to the event of interest or the last follow-up. Patients’ survival was estimated by the Kaplan-Meier method and compared with the log-rank test. The univariate and multivariate Cox proportional hazards regression analysis was used to determine the independent prognostic factors of ASC patients. Subgroup analysis was conducted according to different clinical populations. Statistical analysis was performed with R software (version 4.1.0). All tests are two-tailed, and *P <*0.05 was considered statistically significant.

## 3 Results

### 3.1 Characteristics of Patients

In the SEER database cohort, 1379 eligible cases were enrolled. The mean age of diagnosis was 68.53 ± 9.76 years, 1173 (85.1%) patients underwent lobectomy, and 206 (14.9%) patients underwent sublobar resection. Statistically significant differences appear in terms of age, location, Pathologic T descriptor, Pathologic N descriptor, stage, LND, and chemotherapy. After 1:3 PSM, 463 ASC patients treated with lobectomy (n=337) or sublobar resection (n=126) were confirmed in the analysis. Baseline characteristics were well balanced between the two groups (**Table 1**).

**Table 1:** Baseline characteristics of the SEER cohort before and after PSM.

Characteristics	Before PSM				After PSM			
	Overall	Lobectomy	Sublobar resection	P value	Overall	Lobectomy	Sublobar resection	P value
n	1379	1173	206		463	337	126	
Age(mean ± SD)	68.53 ± 9.76	68.11 ± 9.83	70.97 ± 8.96	<0.001	69.95 ± 9.13	69.94 ± 9.15	69.97 ± 9.10	0.974
Sex (%)				0.740				0.971
Female	638 (46.3)	540 (46.0)	98 (47.6)		245 (52.9)	179 (53.1)	66 (52.4)	
Male	741 (53.7)	633 (54.0)	108 (52.4)		218 (47.1)	158 (46.9)	60 (47.6)	
Race (%)				0.068				0.766
Black	120 (8.7)	104 (8.9)	16 (7.8)		27 (5.8)	19 (5.6)	8 (6.3)	
White	1171 (84.9)	987 (84.1)	184 (89.3)		428 (92.4)	313 (92.9)	115 (91.3)	
Other	88 (6.4)	82 (7.0)	6 (2.9)		8 (1.7)	5 (1.5)	3 (2.4)	
Location (%)				0.008				0.605
Lower lobe	434 (31.5)	383 (32.6)	51 (24.8)		125 (27.0)	95 (28.2)	30 (23.8)	
Middle lobe	59 (4.3)	56 (4.8)	3 (1.5)		7 (1.5)	5 (1.5)	2 (1.6)	
Upper lobe	868 (62.9)	718 (61.2)	150 (72.8)		327 (70.6)	235 (69.7)	92 (73.0)	
Overlapping lesion	18 (1.3)	16 (1.4)	2 (1.0)		4 (0.9)	2 (0.6)	2 (1.6)	
Pathologic T descriptor (%)				<0.001				0.854
T1	501 (36.3)	401 (34.2)	100 (48.5)		217 (46.9)	159 (47.2)	58 (46.0)	
T2	553 (40.1)	474 (40.4)	79 (38.4)		185 (40.0)	133 (39.5)	52 (41.3)	
T3	209 (15.2)	190 (16.2)	19 (9.2)		43 (9.3)	33 (9.8)	10 (7.9)	
T4	116 (8.4)	108 (9.2)	8 (3.9)		18 (3.9)	12 (3.6)	6 (4.8)	
Pathologic N descriptor (%)				<0.001				0.210
N0	954 (69.2)	776 (66.2)	178 (86.4)		399 (86.2)	294 (87.2)	105 (83.3)	
N1	209 (15.2)	207 (17.6)	2 (1.0)		12 (2.6)	10 (3.0)	2 (1.6)	
N2	208 (15.1)	184 (15.7)	24 (11.7)		52 (11.2)	33 (9.8)	19 (15.1)	
N3	8 (0.6)	6 (0.5)	2 (1.0)		–	–	–	
Pathologic TNM stage (%)				<0.001				0.007
I	730 (52.9)	564 (48.1)	166 (80.6)		330 (71.3)	236 (70.0)	94 (74.6)	
II	309 (22.4)	300 (25.6)	9 (4.4)		65 (14.0)	57 (16.9)	8 (6.3)	
III	340 (24.7)	309 (26.3)	31 (15.0)		68 (14.7)	44 (13.1)	24 (19.1)	
LND (%)				<0.001				0.111
No	107 (7.8)	27 (2.3)	80 (38.8)		17 (3.7)	9 (2.7)	8 (6.4)	
Yes	1272 (92.2)	1146 (97.7)	126 (61.2)		446 (96.3)	328 (97.3)	118 (93.6)	
Radiation (%)				0.190				0.444
No	1151 (83.5)	986 (84.1)	165 (80.1)		404 (87.3)	297 (88.1)	107 (84.9)	
Yes	228 (16.5)	187 (15.9)	41 (19.9)		59 (12.7)	40 (11.9)	19 (15.1)	
Chemotherapy (%)				<0.001				0.364
No	937 (68.0)	775 (66.1)	162 (78.6)		378 (81.6)	279 (82.8)	99 (78.6)	
Yes	442 (32.0)	398 (33.9)	44 (21.4)		85 (18.4)	58 (17.2)	27 (21.4)	
Size (mean ± SD, cm)	3.43 ± 1.93	3.63 ± 1.96	2.33 ± 1.26	<0.001	2.89 ± 1.60	3.09 ± 1.68	2.34 ± 1.20	<0.001

SEER, Surveillance, epidemiology, and End Results; PSM, propensity score matching; LND, lymph node dissection.

A total of 466 cases were included in the SPH cohort, with a mean age of diagnosis of 59.52 ± 9.80 years. 405 (86.9%) and 61 (13.1%) patients received lobectomy and sublobar resection, respectively. Significant differences were found in age, Pathologic T descriptor, Pathologic N descriptor, and LND. Also, after 1:3 PSM, 244 patients (183 cases of lobectomy and 61 cases of sublobar resection) were confirmed in the final analysis (**Table 2**).

**Table 2:** Baseline characteristics of the SPH cohort before and after PSM.

Characteristics	Before PSM				After PSM			
	Overall	Lobectomy	Sublobar resection	P value	Overall	Lobectomy	Sublobar resection	P value
n	466	405	61		244	183	61	
Age (mean ± SD)	59.52 ± 9.80	59.09 ± 9.44	62.34 ± 11.61	0.016	60.66 ± 10.88	60.10 ± 10.60	62.34 ± 11.61	0.164
Sex (%)				0.091				1.000
Female	155 (33.3)	141 (34.8)	14 (22.9)		56 (22.9)	42 (22.9)	14 (22.9)	
Male	311 (66.7)	264 (65.2)	47 (77.1)		188 (77.1)	141 (77.1)	47 (77.1)	
Location (%)				0.609				0.959
Lower lobe	122 (26.2)	102 (25.2)	20 (32.8)		74 (30.3)	54 (29.5)	20 (32.8)	
Middle lobe	31 (6.7)	28 (6.9)	3 (4.9)		14 (5.7)	11 (6.0)	3 (4.9)	
Upper lobe	292 (62.7)	257 (63.5)	35 (57.4)		143 (58.6)	108 (59.0)	35 (57.4)	
Overlapping lesion	21 (4.51)	18 (4.4)	3 (4.9)		13 (5.3)	10 (5.5)	3 (4.9)	
Pathologic T descriptor (%)				<0.001				0.556
T1	132 (28.3)	105 (25.9)	27 (44.3)		95 (38.9)	68 (37.2)	27 (44.3)	
T2	179 (38.4)	154 (38.0)	25 (41.0)		114 (46.7)	89 (48.6)	25 (41.0)	
T3	46 (9.9)	37 (9.1)	9 (14.8)		35 (14.3)	26 (14.2)	9 (14.7)	
T4	109 (23.4)	109 (26.9)	0 (0.0)		–	–	–	
Pathologic N descriptor (%)				0.886				0.666
N0	265 (56.9)	229 (56.5)	36 (59.0)		144 (59.0)	108 (59.0)	36 (59.0)	
N1	43 (9.2)	37 (9.1)	6 (9.8)		18 (7.4)	12 (6.6)	6 (9.8)	
N2	158 (33.9)	139 (34.3)	19 (31.2)		82 (33.6)	63 (34.4)	19 (31.2)	
Pathologic TNM stage (%)				0.007				0.665
I	159 (34.1)	128 (31.6)	31 (50.8)		112 (45.9)	81 (44.3)	31 (50.8)	
II	74 (15.9)	64 (15.8)	11 (18.0)		50 (20.5)	39 (21.3)	11 (18.0)	
III	233 (50.0)	213 (52.6)	19 (31.2)		82 (33.6)	63 (34.4)	19 (31.2)	
LND (%)				<0.001				0.057
No	131 (28.1)	101 (24.9)	30 (49.2)		93 (38.1)	63 (34.4)	30 (49.2)	
Yes	335 (71.9)	304 (75.1)	31 (50.8)		151 (61.9)	120 (65.6)	31 (50.8)	
Radiation (%)				0.667				0.881
No	267 (57.3)	230 (56.8)	37 (60.7)		144 (59.0)	107 (58.5)	37 (60.7)	
Yes	199 (42.7)	175 (43.2)	24 (39.3)		100 (41.0)	76 (41.5)	24 (39.3)	
Chemotherapy (%)				0.143				0.485
No	142 (30.5)	118 (29.1)	24 (39.3)		85 (34.8)	61 (33.3)	24 (39.3)	
Yes	324 (69.5)	287 (70.9)	37 (60.7)		159 (65.2)	122 (66.7)	37 (60.7)	
Size (mean ± SD, cm)	3.53 ± 1.81	3.64 ± 1.84	2.79 ± 1.37	0.001	3.13 ± 1.43	3.24 ± 1.43	2.79 ± 1.37	0.031
CEA (mean ± SD, ug/L)	14.52 ± 30.81	14.46 ± 30.60	14.92 ± 32.43	0.913	14.55 ± 29.76	14.42 ± 28.91	14.92 ± 32.43	0.910
CYFRA211 (mean ± SD, ng/ml)	4.01 ± 8.22	4.04 ± 8.18	3.83 ± 8.52	0.855	3.30 ± 5.80	3.13 ± 4.57	3.83 ± 8.52	0.413

SPH, Shanghai Pulmonary Hospital; PSM, propensity score matching; LND, lymph node dissection.

### 3.2 Impact of Surgical Types on Survival Outcomes in ASC Patients

**Figure 2:**
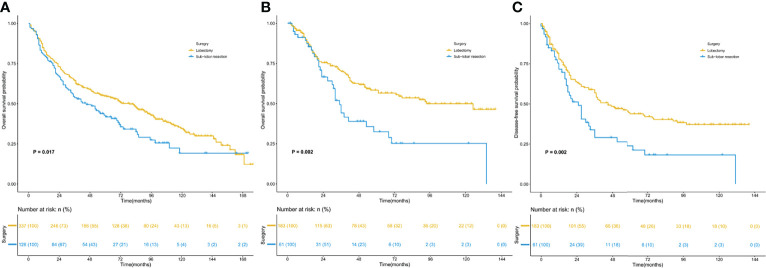
Kaplan–Meier survival curves for patients with lung adenosquamous carcinoma. **(A)** overall survival of the SEER cohort; **(B)** overall survival of the SPH cohort; **(C)** disease-free survival of the SPH cohort. SEER, Surveillance, Epidemiology, and End Results; SPH, Shanghai Pulmonary Hospital.

Survival analysis of the two matched populations (**Figure 2**) showed that the 5-year OS rate of patients who underwent lobectomy was 46.9% in the SEER data cohort. In contrast, those of patients who underwent sublobar resection were 33.3%, indicating a statistical difference in OS (*P* =0.017). In SPH data, statistical differences were observed in both DFS and OS (5-year DFS: 29.5% vs. 14.8%, *P* =0.002; 5-year OS: 35.0% vs. 16.4%, *P* =0.002).

### 3.3 Sublobar Resection as an Adverse Prognostic Factor for Overall Survival in ASC Patients

Univariate and multivariate Cox regression analyses (**Table 3**) showed that sublobar resection was independently associated with worse OS in both SEER and SPH database (SEER: HR: 1.40, 95%CI: 1.08-1.81, *P* =0.012; SPH: HR: 1.73, 95%CI: 1.11-2.70, *P* =0.015). Age, Pathologic N descriptor, carcinoembryonic antigen (CEA), and cytokeratin 19 fragments (CYFRA211) were independent factors for the survival of ASC patients.

**Table 3:** Univariate and multivariate analyses for overall survival.

Characteristics	Univariate analyses		Multivariate analyses	
	HR (95%CI)	P value	HR (95%CI)	P value
**SEER cohort**				
Age	1.03 (1.02-1.05)	<0.001	1.04 (1.02-1.05)	<0.001
Sex(Male)	1.49 (1.18-1.89)	0.001	1.44 (1.14-1.83)	<0.001
Race				
Black	Reference			
White	0.77 (0.48-1.23)	0.273		
Other	0.37 (0.11-1.26)	0.113		
Location				
Lower lobe	Reference			
Middle lobe	0.34 (0.08-1.37)	0.128		
Upper lobe	1.21 (0.93-1.59)	0.157		
Overlapping lesion	1.26 (0.40-4.03)	0.693		
Pathologic T descriptor				
T1	Reference		Reference	
T2	1.69 (1.31-2.17)	<0.001	1.47 (1.13-1.91)	0.004
T3-T4	2.08 (1.47-2.96)	<0.001	2.69 (1.80-4.03)	<0.001
Pathologic N descriptor (Positive)	1.61 (1.18-2.20)	0.003	2.37 (1.60-3.51)	<0.001
Surgery(Sublobar resection)	1.36 (1.05-1.76)	0.018	1.40 (1.08-1.81)	0.012
LND(Yes)	0.55 (0.32-0.94)	0.029	0.61 (0.35-1.05)	0.073
Radiation(Yes)	1.30 (0.93-1.81)	0.125	0.91 (0.58-1.45)	0.705
Chemotherapy(Yes)	1.21 (0.90-1.62)	0.200	0.76 (0.48-1.22)	0.257
**SPH cohort**				
Age	1.02 (1.00-1.04)	0.014	1.03 (1.01-1.05)	0.003
Sex(Male)	1.15 (0.72-1.84)	0.557		
Location				
Lower lobe	Reference			
Middle lobe	1.14 (0.44-2.98)	0.786		
Upper lobe	1.31 (0.84-2.05)	0.239		
Overlapping lesion	0.64 (0.19-2.12)	0.468		
Pathologic T descriptor				
T1	Reference		Reference	
T2	1.46 (0.95-2.24)	0.088	1.13 (0.72-1.78)	0.587
T3	1.71 (0.95-3.07)	0.074	1.27 (0.67-2.41)	0.471
Pathologic N descriptor (Positive)	1.63 (1.11-2.40)	0.013	2.11 (1.16-3.84)	0.015
Surgery(Sublobar resection)	1.95 (1.28-2.96)	0.002	1.73 (1.11-2.70)	0.015
LND(Yes)	1.25 (0.84-1.85)	0.263	0.89 (0.48-1.63)	0.697
Radiation(Yes)	0.98 (0.66-1.44)	0.904	1.16 (0.78-1.73)	0.463
Chemotherapy(Yes)	0.92 (0.60-1.40)	0.696	1.32 (0.82-2.11)	0.253
CEA(≥5.0ug/L)	2.06 (1.39-3.04)	<0.001	1.59 (1.04-2.43)	0.033
CYFRA211(≥2.5ng/ml)	1.96 (1.33-2.90)	0.001	1.76 (1.13-2.74)	0.013

HR, hazard ratio; CI, confidence interval; LND, lymph node dissection.

### 3.4 Survival Outcomes of Lobectomy and Sublobar Resection in Stage I ASC

**Figure 3:**
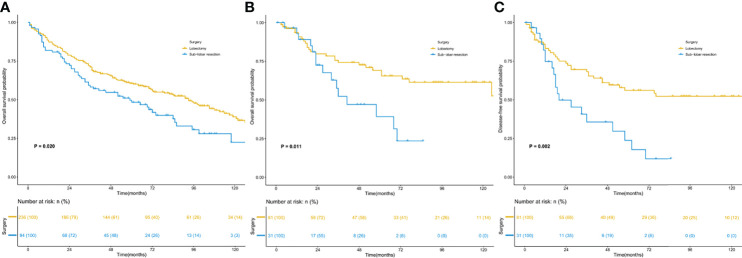
Kaplan–Meier survival curves for patients with stage I. **(A)** overall survival of the SEER cohort; **(B)** overall survival of the SPH cohort; **(C)** disease-free survival of the SPH cohort. SEER, Surveillance, Epidemiology, and End Results; SPH, Shanghai Pulmonary Hospital.

Survival analysis (**Figure 3**) was conducted for the most common stage I ASC, with 300 patients in the SEER database and 112 patients in the SPH database. The analysis results proved that in both the SEER database and SPH database, the OS of patients receiving lobectomy was better than that receiving sublobar resection (SEER: 5-year OS: 50.4% vs. 39.4%, *P* =0.020; SPH: 5-year OS: 46.9% vs. 16.1%, *P* =0.011). In addition, the DFS benefit was highlighted in SPH patients with lobectomy (5-year DFS: 39.5% vs. 12.9%, *P* =0.002).

### 3.5 Subgroup Analysis

**Figure 4:**
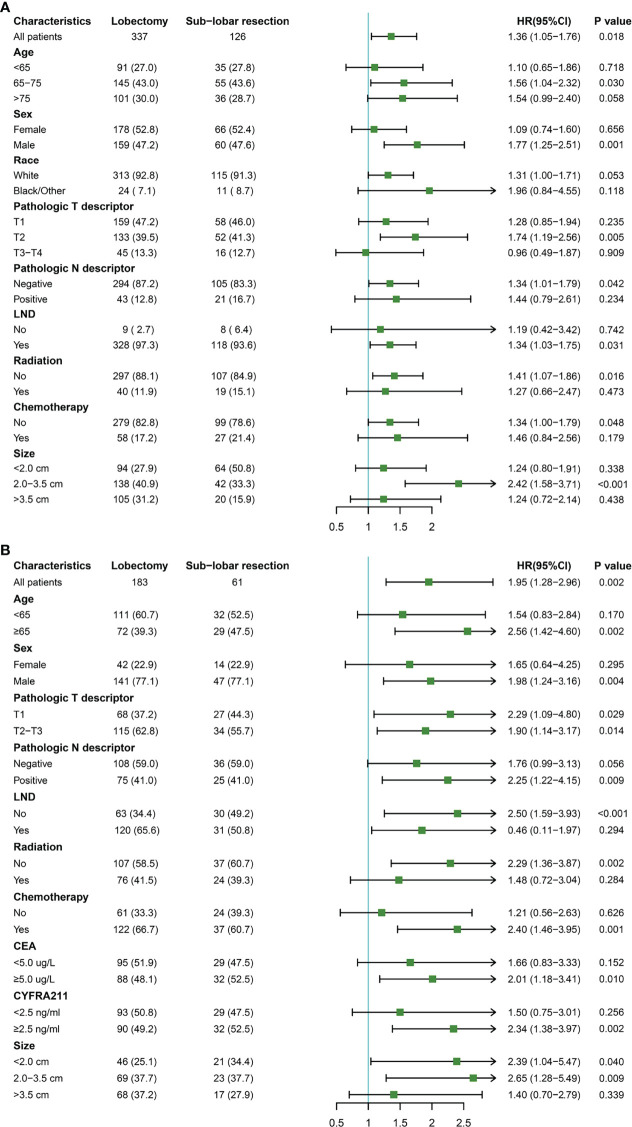
Subgroup analysis for lung adenosquamous carcinoma patients according to surgery type. **(A)** the Surveillance, Epidemiology, and End Results cohort; **(B)** the Shanghai Pulmonary Hospital cohort.

Further investigation was conducted on the survival benefits of lobectomy and sublobar resection in different population subgroups (**Figure 4**). The results showed that when the tumor size was 2.0-3.5cm in the SEER database, lobectomy was superior to sublobar resection (*P <*0.001). But there was no significant difference in survival between the patients with tumor size <2.0cm and >3.5cm (*P* =0.338 for size <2.0cm; *P* =0.438 for size >3.5cm). In the analysis of SPH data, except for tumor size >3.5cm group, tumor size ≤3.5cm with lobectomy was observed better OS rates (P =0. 040 for size < 2.0cm; P =0.009 for size was 2.0-3.5cm; *P* =0.339 for size >3.5cm). It was also noticed that all of the ASC patient subtypes tended to benefit more from lobectomy than sublobar resection, especially in patients who were elderly, male, T2, with or without LND, no radiation, CEA ≥5.0ug/L, and CYFRA211 ≥2.5ng/ml.

## 4 Discussion

In recent years, an increasing number of studies have focused on reducing the excision range of surgical resection to ensure the maximum benefit for NSCLC patients. As far as we know, almost no study has been carried out to compare the survival benefit of lobectomy versus sublobar resection in ASC with a poor prognosis. In this study, we conducted a double demonstration by using the SEER database and SPH database. The results showed that lobectomy was associated with better OS and DFS benefits than sublobar resection for ASC patients.

As a subtype of lung cancer with higher malignancy, ASC has a lower survival rate than other types of NSCLC (3, 4, 12–14). Gawrychowski et al. (14) retrospectively analyzed 96 patients with ASC who underwent radical surgery and found that the postoperative 5-year and 10-year cumulative survival rates were 25.4% and 19.2%, respectively. In addition, the 5-year cumulative survival rate of pathological stage IA was 63.3%. Maeda and colleagues (4) noted that the 5-year survival rate for all stages was 23.3% in 114 ASC patients, the 5-year survival rate of stage IA was 42.0%, and 19.3% for stage IB. Another earlier study (3) analyzed 872 patients of the SEER database diagnosed with ASC between 1988 and 2002 found a 5-year survival rate of 59.4% for stage I patients after lobectomy. In this study, we conducted survival analysis for lobectomy and sublobar resection, separately. It is found that the 5-year OS rate of all stages patients in the SEER database was 46.9% and 33.3%, respectively. While in the SPH data, the 5-year OS of lobectomy and sublobar resection was 35.0% and 16.4%, and the 5-year DFS was 29.5% and 14.8%. Similar results were observed in stage I patients. The difference in survival rate between the two cohorts is likely due to the difference in TNM stage and race composition of the ASC patients enrolled in surgery.

Lobectomy has been considered the standard surgical procedure for early-stage NSCLC in the past decade (8). However, a growing number of researches have challenged this conclusion. Some surgeons (7, 15–17) recommended sublobar resection as a priority for patients who are older, have impaired lung function, or are at higher risk of surgery. On the other hand, several studies (18–21) have extended the indications for surgery to patients with generally good lung function and younger age. Cao et al. (22) conducted a meta-analysis of 54 studies and found that lobectomy patients did not show any significant differences in OS or DFS between those who intentionally selected sublobar resection. A prospective randomized controlled trial (JCOG0802/WJOG4607L) was conducted in Japan enrolled 554 lobectomy patients and 552 segmentectomy patients (23). Its latest follow-up results showed that the 5-year OS rate of segmentectomy was not worse than that of lobectomy in tumor size ≤2 cm NSCLC patients (94.3% vs. 91.1%; HR: 0.663, 95% CI: 0.474-0.927; one-sided *P*<0.0001 for non-inferiority; *P*=0.0082 for superiority), and the 5-year relapse-free survival had no significant difference (88.0% vs. 87.9%; HR: 0.998, 95% CI: 0.753-1.323; *P*=0.9889). In addition, segmentectomy had a better protective effect on lung function (24). However, some scholars raised objections (25–27). Subramanian et al. (27) conducted a retrospective analysis of stage IA NSCLC patients in the National Cancer Data Base (NCDB). They found that although the OS of patients underwent lobectomy and sublobar resection were similar, sublobar resection was associated with a 39% increased risk of recurrence. Khullar et al. (25) found that clinical stage IA NSCLC with wedge and segmental resection showed significantly worse OS than lobectomy.

Surgical options for ASC have rarely been reported. A study involving 114 ASC patients observed that lobectomy was a favorable prognostic factor (HR: 0.750, 95%CI: 0.613-0.917, P=0.005) (4). However, the study did not analyze the prognostic value of sublobar resection. Our study proved that lobectomy could bring better benefits in OS and DFS compared with sublobar resection, and sublobar resection was an adverse prognostic factor for ASC patients. We further analyzed stage I patients in the SEER and SPH databases and found that the OS of patients who received lobectomy was better than that of sublobar resection. DFS benefits of lobectomy also were observed in SPH patients.

As for ASC patients, they can obtain a longer survival time from lobectomy. It is possible that a broader range of tumor resection reduces the number of malignant cancer cells that cannot be inhibited or killed by postoperative adjuvant therapy, at the same time avoid the existence of potential micrometastasis. It thus reduces the possibility of postoperative lymph node metastasis and blood metastasis that are common in ASC. In subgroup analysis, there is a tendency to benefit more from lobectomy than sublobar resection, even though no difference in survival was observed in patients with other than 2.0-3.5 cm tumor size in the SEER database and patients with > 3.5cm tumor size in the SPH database. Regardless of tumor size, sublobar resection is used to replace traditional lobectomy for ASC, a particular subtype of NSCLC, which requires extreme caution. Subgroup analysis also showed that lobectomy was the first choice for the elderly, male, T2, with or without LND, no radiation, CEA≥5.0ug/L, and CYFRA211≥2.5ng/ml.

As a retrospective study, this research may be affected by unmeasured confounding factors. Even though we have thoroughly analyzed and compared the two databases and used PSM to balance most of the confounding factors that affect the comparison of surgical methods, the lack of information on preoperative health status and comorbidities in the SEER database may lead to biases in treatment selection. In addition, resection margin is considered to be an essential factor affecting the prognosis of NSCLC after sublobar resection. Because of the retrospective nature of this study, we are unable to examine the association between the margin and the survival result. Prospective clinical trials with large sample sizes are needed to improve our understanding of surgical options for ASC.

In conclusion, the results of this study suggest that lobectomy patients obtain more survival benefits than sublobar resection for more aggressive ASC. Lobectomy remains the first option for ASC patients, included stage I. Well-designed prospective trials are still needed to verify this conclusion in the future.

## Data Availability Statement

The raw data supporting the conclusions of this article will be made available by the authors, without undue reservation.

## Author Contributions

All authors participated in manuscript writing and approved the final version of the manuscript. SZ and TG conceived and designed the analysis. Collection and assembly of data were performed by SZ, TG, YX, and DZ. Analysis and interpretation of the data were supported by SZ, TG, LS, and JZ. NS and PZ conducted a critical review of the manuscript, contributing important intellectual content. All authors contributed to the article and approved the submitted version.

## Funding

This work was supported by Shanghai Science and Technology Committee (No. 19XD1423200), Clinical Research foundation of Shanghai Pulmonary Hospital (FKLY20004), and the Clinical Research Plan of Shanghai Hospital Development Center (No. SHDC2020CR2020B), the Fundamental Research Funds for the Central Universities (No. 22120180510).

## Conflict of Interest

The authors declare that the research was conducted in the absence of any commercial or financial relationships that could be construed as a potential conflict of interest.

## Publisher’s Note

All claims expressed in this article are solely those of the authors and do not necessarily represent those of their affiliated organizations, or those of the publisher, the editors and the reviewers. Any product that may be evaluated in this article, or claim that may be made by its manufacturer, is not guaranteed or endorsed by the publisher.
